# *catena*-Poly[[trimethyl­tin(IV)]-μ-2,5-difluoro­benzoato-κ^2^ *O*:*O*′]

**DOI:** 10.1107/S1600536808040774

**Published:** 2008-12-10

**Authors:** Liyuan Wen, Handong Yin, Wenkuan Li, Daqi Wang

**Affiliations:** a College of Chemistry and Chemical Engineering, Liaocheng University, Shandong 252059, People’s Republic of China

## Abstract

In the title polymeric coordination compound, [Sn(CH_3_)_3_(C_7_H_3_F_2_O_2_)]_*n*_, the Sn atom exhibits a distorted trigonal-bipyramidal coordination geometry with the carboxyl­ate O atoms in the axial positions and the equatorial positions occupied by the methyl groups. The two Sn—O bond lengths are 2.225 (5) and 2.410 (6) Å.

## Related literature

For a related structure, see: Wang *et al.* (2007 ▶).
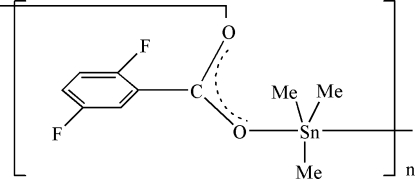


## Experimental

### 

#### Crystal data

[Sn(CH_3_)_3_(C_7_H_3_F_2_O_2_)]
*M* *_r_* = 320.91
Tetragonal, 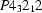

*a* = 9.8857 (9) Å
*c* = 24.896 (2) Å
*V* = 2433.0 (4) Å^3^
*Z* = 8
Mo *K*α radiation
μ = 2.11 mm^−1^
*T* = 298 (2) K
0.38 × 0.29 × 0.27 mm

#### Data collection

Siemens SMART CCD diffractometer
Absorption correction: multi-scan (*SADABS*; Sheldrick, 1996 ▶) *T*_min_ = 0.502, *T*_max_ = 0.600 (expected range = 0.474–0.566)
10078 measured reflections
2152 independent reflections
1996 reflections with *I* > 2σ(*I*)
*R*_int_ = 0.034

#### Refinement

*R*[*F* ^2^ > 2σ(*F* ^2^)] = 0.036
*wR*(*F* ^2^) = 0.107
*S* = 1.00
2152 reflections
136 parameters
H-atom parameters constrained
Δρ_max_ = 0.54 e Å^−3^
Δρ_min_ = −0.46 e Å^−3^
Absolute structure: Flack (1983 ▶), 830 Friedel pairs
Flack parameter: −0.01 (8)

### 

Data collection: *SMART* (Siemens, 1996 ▶); cell refinement: *SAINT* (Siemens, 1996 ▶); data reduction: *SAINT*; program(s) used to solve structure: *SHELXS97* (Sheldrick, 2008 ▶); program(s) used to refine structure: *SHELXL97* (Sheldrick, 2008 ▶); molecular graphics: *SHELXTL* (Sheldrick, 2008 ▶); software used to prepare material for publication: *SHELXTL*.

## Supplementary Material

Crystal structure: contains datablocks I, global. DOI: 10.1107/S1600536808040774/gk2177sup1.cif

Structure factors: contains datablocks I. DOI: 10.1107/S1600536808040774/gk2177Isup2.hkl

Additional supplementary materials:  crystallographic information; 3D view; checkCIF report

## Figures and Tables

**Table:** 

Sn1—C9	2.103 (9)
Sn1—C10	2.110 (8)
Sn1—C8	2.118 (7)
Sn1—O1	2.225 (5)
Sn1—O2^i^	2.410 (6)

**Table:** 

O1—Sn1—O2^i^	174.15 (19)

Symmetry code: (i) 
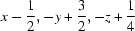
.

**Fig. 1.**
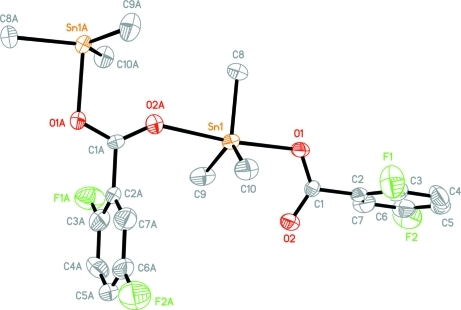
The molecular structure of the compound, showing 50% probability displacement ellipsoids. H atoms have been omitted for clarity. Symmetry codes: (A) = x - 1/2,-y + 3/2,-z + 1/4, (B) = x + 1/2,-y + 3/2,-z + 1/4

## supplementary crystallographic information

### Comment

In recent years the organotin derivatives have attracted considerable attention due to a significant antimicrobial properties as well as antitumor activities. Studies on organotin complexes containing carboxylate ligands with an additional donor atom (e.g N, S or F) that is available for coordinating to Sn atom have revealed that new structural types may lead to different activities. We have therefore synthesized the title compound, and present its crystal structure here. The molecular structure of the compound is shown in Fig.1 The Sn atom, assumes a distorted trigonal bipyramidal coordination geometry, provided by three methyl groups at the equatorial positions and two carboxylate groups at the axial positions. The Sn—O bond lengths in the compound (Table 1), are similar to those found in related organotin carboxylates (Wang *et al.*, 2007). In the crystal packing, molecules are linked by intermolecular C—H···F hydrogen bonds (Fig.2, Table 1,)

### Experimental

The reaction was carried out under nitrogen atmosphere. 2,5-Difluorobenzoic acid (1 mmol) and sodium ethoxide (1.2 mmol) were added to a stirred solution of benzene (30 ml) in a Schlenk flask and stirred for 0.5 h. Trimethyltin chloride (1 mmol) was then added to the reactor and the reaction mixture was stirred for 12 h at room temperature. The resulting clear solution was evaporated under vacuum. The product was crystallized from dichloromethane/methanol (1:1) to yield colourless block crystals (yield 83%. m.p.393K). Anal. Calcd (%) for C_10_H_12_F_2_O_2_Sn(Mr = 320.91): C, 37.43; H, 3.77; F, 11.84; Sn, 36.99. Found (%): C, 37.39; H, 3.86; F, 11.78; Sn, 36.89.

### Refinement

The H atoms were positioned geometrically, with methyl C—H distances of 0.96 Å and aromatic C—H distances of 0.93 Å, and refined as riding on their parent atoms, with *U*_iso_(H) = 1.2 *U*_eq_(C, O) or 1.5 *U*_eq_(C) for the methyl group.

### Crystal data

**Table:**

[Sn(CH_3_)_3_(C_7_H_3_F_2_O_2_)]	*D*_x_ = 1.752 Mg m^−^^3^
*M**_r_* = 320.91	Mo *K*α radiation, λ = 0.71073 Å
Tetragonal, *P*4_3_2_1_2	Cell parameters from 5675 reflections
Hall symbol: P 4nw 2abw	θ = 2.2–26.4°
*a* = 9.8857 (9) Å	µ = 2.11 mm^−^^1^
*c* = 24.896 (2) Å	*T* = 298 K
*V* = 2433.0 (4) Å^3^	Block, colourless
*Z* = 8	0.38 × 0.29 × 0.27 mm
*F*(000) = 1248	

### Data collection

**Table:**

Siemens SMART CCD diffractometer	2152 independent reflections
Radiation source: fine-focus sealed tube	1996 reflections with *I* > 2σ(*I*)
graphite	*R*_int_ = 0.034
φ and ω scans	θ_max_ = 25.0°, θ_min_ = 2.2°
Absorption correction: multi-scan (*SADABS*; Sheldrick, 1996)	*h* = −11→11
*T*_min_ = 0.502, *T*_max_ = 0.600	*k* = −11→7
10078 measured reflections	*l* = −16→29

### Refinement

**Table:**

Refinement on *F*^2^	Secondary atom site location: difference Fourier map
Least-squares matrix: full	Hydrogen site location: inferred from neighbouring sites
*R*[*F*^2^ > 2σ(*F*^2^)] = 0.036	H-atom parameters constrained
*wR*(*F*^2^) = 0.107	*w* = 1/[σ^2^(*F*_o_^2^) + (0.066*P*)^2^ + 4.861*P*] where *P* = (*F*_o_^2^ + 2*F*_c_^2^)/3
*S* = 1.00	(Δ/σ)_max_ < 0.001
2152 reflections	Δρ_max_ = 0.54 e Å^−^^3^
136 parameters	Δρ_min_ = −0.45 e Å^−^^3^
0 restraints	Absolute structure: Flack (1983)
Primary atom site location: structure-invariant direct methods	Flack parameter: −0.01 (8)

### Fractional atomic coordinates and isotropic or equivalent isotropic displacement parameters (Å^2^)

**Table:**

	*x*	*y*	*z*	*U*_iso_*/*U*_eq_	
Sn1	0.79786 (5)	0.82634 (5)	0.11522 (2)	0.05163 (18)	
F1	1.1717 (7)	1.1333 (7)	0.1597 (3)	0.111 (2)	
F2	1.4123 (8)	1.0943 (8)	−0.0335 (3)	0.131 (3)	
O1	0.9583 (5)	0.9696 (5)	0.0890 (2)	0.0632 (14)	
O2	1.1085 (6)	0.8095 (6)	0.1065 (3)	0.0786 (18)	
C1	1.0763 (7)	0.9249 (7)	0.0922 (3)	0.0552 (19)	
C2	1.1900 (8)	1.0242 (7)	0.0773 (3)	0.0561 (18)	
C3	1.2313 (10)	1.1239 (11)	0.1101 (4)	0.080 (3)	
C4	1.3367 (11)	1.2144 (9)	0.0992 (5)	0.089 (3)	
H4	1.3649	1.2787	0.1240	0.107*	
C5	1.3953 (9)	1.2021 (11)	0.0498 (5)	0.082 (3)	
H5	1.4635	1.2615	0.0395	0.098*	
C6	1.3539 (10)	1.1028 (11)	0.0153 (4)	0.081 (3)	
C7	1.2516 (10)	1.0141 (11)	0.0269 (4)	0.078 (3)	
H7	1.2240	0.9495	0.0020	0.093*	
C8	0.6502 (9)	0.9694 (8)	0.0912 (3)	0.067 (2)	
H8A	0.6849	1.0227	0.0621	0.100*	
H8B	0.6287	1.0274	0.1210	0.100*	
H8C	0.5700	0.9229	0.0797	0.100*	
C9	0.8458 (10)	0.6721 (11)	0.0603 (4)	0.081 (3)	
H9A	0.7645	0.6261	0.0497	0.122*	
H9B	0.9065	0.6088	0.0769	0.122*	
H9C	0.8882	0.7108	0.0292	0.122*	
C10	0.8626 (9)	0.8328 (10)	0.1959 (3)	0.070 (2)	
H10A	0.9112	0.7514	0.2043	0.105*	
H10B	0.7854	0.8405	0.2191	0.105*	
H10C	0.9207	0.9095	0.2011	0.105*	

### Atomic displacement parameters (Å^2^)

**Table:**

	*U*^11^	*U*^22^	*U*^33^	*U*^12^	*U*^13^	*U*^23^
Sn1	0.0368 (3)	0.0477 (3)	0.0704 (3)	0.0054 (2)	−0.0009 (2)	0.0029 (2)
F1	0.121 (6)	0.108 (5)	0.103 (4)	−0.027 (4)	0.039 (4)	−0.022 (4)
F2	0.138 (6)	0.136 (6)	0.120 (5)	0.010 (5)	0.067 (5)	0.039 (4)
O1	0.040 (3)	0.047 (3)	0.102 (4)	0.009 (2)	0.009 (3)	0.020 (3)
O2	0.054 (3)	0.054 (3)	0.128 (5)	0.005 (3)	−0.001 (3)	0.029 (4)
C1	0.031 (3)	0.047 (4)	0.087 (5)	−0.004 (3)	0.012 (3)	0.014 (4)
C2	0.033 (3)	0.046 (4)	0.089 (5)	0.005 (3)	0.003 (4)	0.015 (4)
C3	0.063 (6)	0.083 (7)	0.094 (7)	0.012 (5)	0.020 (5)	0.004 (6)
C4	0.100 (8)	0.048 (5)	0.119 (8)	−0.007 (5)	0.014 (6)	0.007 (5)
C5	0.046 (5)	0.067 (6)	0.133 (9)	0.001 (5)	0.013 (5)	0.035 (6)
C6	0.058 (5)	0.087 (7)	0.099 (7)	0.012 (5)	0.025 (5)	0.039 (6)
C7	0.067 (6)	0.089 (7)	0.077 (6)	0.006 (5)	0.011 (5)	0.026 (5)
C8	0.062 (5)	0.050 (5)	0.088 (5)	0.013 (4)	−0.004 (4)	0.007 (4)
C9	0.074 (6)	0.092 (7)	0.078 (6)	0.025 (6)	−0.012 (5)	−0.009 (5)
C10	0.068 (5)	0.074 (6)	0.068 (5)	0.008 (4)	−0.003 (4)	−0.004 (4)

### Geometric parameters (Å, °)

**Table:**

Sn1—C9	2.103 (9)	C4—H4	0.9300
Sn1—C10	2.110 (8)	C5—C6	1.366 (15)
Sn1—C8	2.118 (7)	C5—H5	0.9300
Sn1—O1	2.225 (5)	C6—C7	1.369 (14)
Sn1—O2^i^	2.410 (6)	C7—H7	0.9300
F1—C3	1.373 (11)	C8—H8A	0.9600
F2—C6	1.349 (11)	C8—H8B	0.9600
O1—C1	1.250 (9)	C8—H8C	0.9600
O2—C1	1.237 (9)	C9—H9A	0.9600
O2—Sn1^ii^	2.410 (6)	C9—H9B	0.9600
C1—C2	1.537 (10)	C9—H9C	0.9600
C2—C3	1.344 (13)	C10—H10A	0.9600
C2—C7	1.399 (12)	C10—H10B	0.9600
C3—C4	1.399 (14)	C10—H10C	0.9600
C4—C5	1.365 (14)		
			
C9—Sn1—C10	125.0 (4)	C6—C5—H5	119.9
C9—Sn1—C8	117.1 (4)	F2—C6—C5	118.8 (10)
C10—Sn1—C8	117.2 (3)	F2—C6—C7	117.7 (11)
C9—Sn1—O1	96.3 (3)	C5—C6—C7	123.4 (10)
C10—Sn1—O1	92.5 (3)	C6—C7—C2	117.7 (11)
C8—Sn1—O1	89.1 (3)	C6—C7—H7	121.2
C9—Sn1—O2^i^	87.8 (3)	C2—C7—H7	121.2
C10—Sn1—O2^i^	88.5 (3)	Sn1—C8—H8A	109.5
C8—Sn1—O2^i^	85.4 (3)	Sn1—C8—H8B	109.5
O1—Sn1—O2^i^	174.15 (19)	H8A—C8—H8B	109.5
C1—O1—Sn1	114.9 (4)	Sn1—C8—H8C	109.5
C1—O2—Sn1^ii^	142.9 (5)	H8A—C8—H8C	109.5
O2—C1—O1	125.8 (6)	H8B—C8—H8C	109.5
O2—C1—C2	118.1 (6)	Sn1—C9—H9A	109.5
O1—C1—C2	116.2 (6)	Sn1—C9—H9B	109.5
C3—C2—C7	117.7 (8)	H9A—C9—H9B	109.5
C3—C2—C1	123.0 (8)	Sn1—C9—H9C	109.5
C7—C2—C1	119.3 (8)	H9A—C9—H9C	109.5
C2—C3—F1	117.8 (9)	H9B—C9—H9C	109.5
C2—C3—C4	125.3 (9)	Sn1—C10—H10A	109.5
F1—C3—C4	116.8 (10)	Sn1—C10—H10B	109.5
C5—C4—C3	115.7 (10)	H10A—C10—H10B	109.5
C5—C4—H4	122.1	Sn1—C10—H10C	109.5
C3—C4—H4	122.1	H10A—C10—H10C	109.5
C4—C5—C6	120.1 (9)	H10B—C10—H10C	109.5
C4—C5—H5	119.9		
			
C9—Sn1—O1—C1	−58.5 (6)	C1—C2—C3—F1	2.4 (13)
C10—Sn1—O1—C1	67.1 (6)	C7—C2—C3—C4	−3.8 (15)
C8—Sn1—O1—C1	−175.6 (7)	C1—C2—C3—C4	178.1 (9)
Sn1^ii^—O2—C1—O1	−166.0 (7)	C2—C3—C4—C5	3.6 (16)
Sn1^ii^—O2—C1—C2	13.5 (15)	F1—C3—C4—C5	179.3 (8)
Sn1—O1—C1—O2	2.8 (12)	C3—C4—C5—C6	−2.5 (15)
Sn1—O1—C1—C2	−176.8 (5)	C4—C5—C6—F2	179.0 (9)
O2—C1—C2—C3	−103.1 (10)	C4—C5—C6—C7	2.0 (16)
O1—C1—C2—C3	76.4 (11)	F2—C6—C7—C2	−179.2 (8)
O2—C1—C2—C7	78.8 (11)	C5—C6—C7—C2	−2.1 (15)
O1—C1—C2—C7	−101.7 (9)	C3—C2—C7—C6	2.9 (13)
C7—C2—C3—F1	−179.5 (8)	C1—C2—C7—C6	−178.9 (8)

Symmetry codes: (i) *x*−1/2, −*y*+3/2, −*z*+1/4; (ii) *x*+1/2, −*y*+3/2, −*z*+1/4.

### Hydrogen-bond geometry (Å, °)

**Table:**

*D*—H···*A*	*D*—H	H···*A*	*D*···*A*	*D*—H···*A*
C5—H5···F1^iii^	0.93	2.63	3.336 (13)	133

Symmetry codes: (iii) *x*+1/2, −*y*+5/2, −*z*+1/4.
